# A 28 GHz GaN 6-Bit Phase Shifter MMIC with Continuous Tuning Calibration Technique

**DOI:** 10.3390/s24041087

**Published:** 2024-02-07

**Authors:** Soyeon Seo, Jinho Lee, Yongho Lee, Hyunchol Shin

**Affiliations:** 1 Department of Electronic Convergence Engineering, Kwangwoon University, Seoul 01897, Republic of Korea; lullaby1014@kw.ac.kr (S.S.); dldyd91@kw.ac.kr (Y.L.); 2 Now with Samsung Electronics Co., Suwon 16677, Republic of Korea; jinh0.lee@samsung.com

**Keywords:** phase shifter, phase calibration technique, gallium nitride (GaN), high electron mobility transistor (HEMT), 28 GHz

## Abstract

A 28 GHz digitally controlled 6-bit phase shifter with a precision calibration technique in GaN high-electron mobility transistor (HEMT) technology is presented for Ka-band phased-array systems and applications. It comprises six stages, in which stages 1 and 2 for 5.625° and 11.25° are designed in the form of a switched-line circuit, and stages 3, 4, and 5 for 22.5°, 45°, and 90° are designed in the form of a switched-filter circuit. The final stage 6 for 180° is designed in a single-to-differential balun followed by a single-pole double-throw (SPDT) switch for achieving an efficient phase inversion. A novel continuous tuning calibration technique is proposed to improve the phase accuracy. It controls the gate bias voltage of off-state HEMTs at the stage 6 SPDT switch for fine calibration of the output phase. Fabricated in a 0.15 μm GaN HEMT process using a die size of 1.75 mm^2^, the circuit produces 64 phase states at 28 GHz with a 5.625° step. The experimental results show that the Root-Mean-Square (RMS) phase error is significantly improved from 8.56° before calibration to 1.08° after calibration. It is also found that the calibration does not induce significant changes for other performances such as the insertion loss, RMS amplitude error, and input-referred P_1dB_. This work successfully demonstrates that the GaN technology can be applied to millimeter-wave high-power phased-array transceiver systems.

## 1. Introduction

The millimeter-wave frequency band at the Ka band is adopted for various wireless applications such as the fifth-generation (5G) frequency range 2 (FR2) cellular network, local-area network, personal-area network, short-range and proximity link system, radar and remote sensing system, etc. This band suffers from significant path loss and attenuation when the link is set up over a long distance or blocked by structural obstacles. To mitigate this problem, a phased-array beamforming transceiver system is highly needed for guaranteeing the wanted directivity and high gain [1]. For realizing such a Ka-band phased-array transceiver system, a precise and high-resolution phase shifter in the form of a monolithic microwave-integrated circuit (MMIC) is needed.

Previous publications show that the digitally controlled phase shifter MMICs have been developed in various semiconductor technologies. Refs. [2,3] presented CMOS digital phase shifters, in which [2] showed a 28 GHz 3-bit phase shifter with a 7° phase error and [3] showed a 27–42 GHz 5-bit phase shifter with a 3.8° phase error. In GaAs technology, Ref. [4] reported a 38 GHz 4-bit phase shifter with a 9.7° phase error, and [5] reported a 31–40 GHz 5-bit phase shifter with a 4.7° phase error.

Meanwhile, in GaN technology, Refs. [6,7,8] reported a 5-bit phase shifter with a 6.4° phase error, a 5-bit phase shifter with a 2.5° phase error, and a 3-bit phase shifter with a 3° phase error, all in the X band. The most recent state-of-the-art works are reported in [9,10]. Kim et al. reported a 6-bit GaN phase shifter MMIC operating at 36–39 GHz [9]. All six circuit stages including the 180°-shifting stage were designed in a low-pass T-type switched filter structure. They reported an RMS phase error of <4.6°, and no phase calibration technique was adopted. Song et al. reported a 6-bit GaN phase shifter MMIC operating at 37–40 GHz [10]. Five circuits stages, except for the 180°-shifting stage, were designed using a low-pass T-type switched filter, too. The 180°-shifting stage consists of two 90° phase shifters which were arranged in a series. They achieved an RMS phase error of <5.36° without any phase calibration technique. Meanwhile, it is also interesting to note that a much simpler 1-bit GaN phase shifter in the X band reported in [11,12] also exhibited the phase errors of 3–5°.

Thus, we can find that the previous digital phase shifter MMICs in any semiconductor process such as CMOS, GaAs or GaN have demonstrated the typical phase error of 3–9°. We note that the phase error can be improved by adopting a phase calibration technique.

The most conventional phase calibration technique is to utilize the least significant bit (LSB). This technique utilizes the LSB stage among the multi-bit circuit stages for the fine phase-tuning purpose [13,14,15], which is referred to as the LSB code calibration technique. It can reduce the phase error by the LSB phase value, but the phase-tuning resolution will be inevitably lowered by 1 bit. For example, in a 6-bit phase shifter, if the LSB code for the 5.625° stage is utilized for the LSB code calibration, the phase-tuning resolution of the overall phase shifter will be reduced from 6-bit to 5-bit; thus, the minimum phase-tuning step will become 11.25° rather than 5.625°.

We can find several CMOS digital phase shifters that employ the LSB code calibration technique. Ref. [13] realized a 7-bit CMOS phase shifter in the X band and utilized the LSB calibration to improve the phase error from 8° to 6° while resulting in the actual phase shift resolution of a 6-bit. Ref. [14] realized an 8-bit GaAs phase shifter in 2.4–4 GHz and utilized the LSB calibration to improve the phase error from 3.5° to 1.5° while resulting in 7-bit phase shift resolution. Ref. [15] realized a 9-bit CMOS phase shifter in 28 GHz and interestingly utilized 3 LSBs for calibration to improve the phase error to 3.5° while resulting in 6-bit phase shift resolution. However, the LSB code calibration technique has disadvantages: for example, the phase-tuning resolution must be lowered by 1 bit and an additional die area overhead is needed for the circuit stage for the LSB code calibration.

In this work, we propose a novel continuous-tuning calibration technique for the digital phase shifter. It effectively overcomes the disadvantages of the conventional LSB code calibration technique and offers very precise and efficient phase error improvement. Also, we apply this calibration technique to a GaN Ka band phase shifter rather than a CMOS. A 28 GHz digitally controlled 6-bit phase shifter MMIC is presented in 0.15 μm GaN HEMT technology. By employing the proposed calibration technique, we successfully demonstrate that the RMS phase error is improved from 8.56° to 1.08° without a noticeable performance degradation in other circuit performance parameters.

## 2. Circuit Design

**Figure 1:**
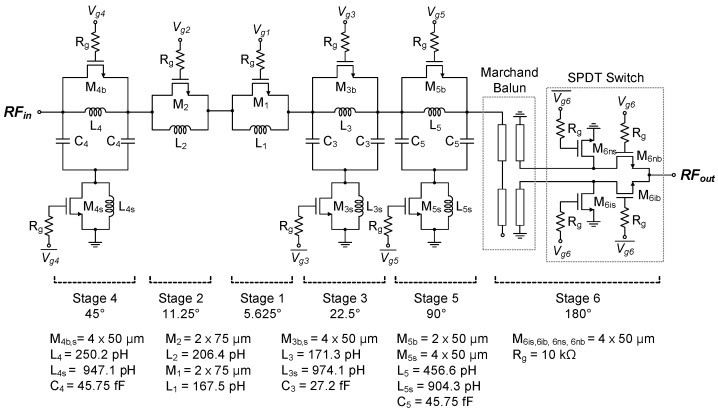
The proposed 6-bit GaN phase shifter MMIC.

Figure 1 shows the circuit schematic of the proposed phase shifter circuit. It comprises six circuit stages, producing 64 phase states from 0° to 360° with a minimum step of 5.625°. Stage 1 for a 5.625° shift and stage 2 for a 11.25° shift are based on the switched-line circuit. Stage 3 for a 22.5° shift, stage 4 for a 45° shift, and stage 5 for a 90° shift are based on the switched-filter circuit. The bypass switch FETs of M_1_, M_2_, M_3b,_ M_4b,_ M_5b_ and the shunt grounding switch FETs of M_3s,_ M_4s,_ M_5s_ are switched to proper on/off states depending on the wanted phase shift value.

For stages 1 and 2, when the bypass switch is off, the signal passes with a certain amount of phase shift induced by the series inductors L_1_ and L_2_. And when the bypass switch is on, the signal passes with no phase shift. For stages 3, 4, and 5, when the bypass switches are off and shunt-grounding switches are on, the π-type low-pass filter is activated and induces a certain amount of phase shift. In contrast, when the bypass switches are on and shunt-grounding switches are off, the signal is directly passed through the bypass switches while the π-type low-pass filter is deactivated.

The final stage 6 for a 180° shift is designed in the form of a Marchand balun [16] followed by a single-pole double-throw (SPDT) switch. It steers the signal into either the inverting (180°) or non-inverting (0°) path, producing the 180° phase shift. It should be pointed out that the gate bias voltage $V_{g6}$ for M_6nb_ and M_6is_ and $\bar{V_{g6}}$ for M_6ib_ and M_6ns_ are tuned for the continuous phase-tuning calibration. The detailed design parameters of the devices are also presented in Figure 1.

In the digital phase shifter, the gate voltages V_g1_–V_g6_ for the switch FETs are set to either 0 V for the on state or −5 V for the off state. The resistors R_g_ of 10 kΩ are adopted to protect the FET’s gate nodes as well as suppress any unwanted RF signal leakage.

The gate widths of the switch FETs are carefully designed by examining the overall phase-shift performance while not inducing significant degradation of the insertion loss. When the FET width is large, its insertion loss can be desirably reduced, whereas its phase shift response can be adversely affected due to the FET’s increased parasitic capacitance. Hence, in this design, we maximize the FET width to minimize the insertion loss while at the same time not seriously degrading the phase shift characteristics.

For the lower-bit stages 1 and 2, M_1,2_ have the total gate width of 150 μm with a unit finger width of 75 μm and two fingers, whose insertion loss is found to be 1.17 dB. For the upper-bit stages 3–6, M_3-6_ have a wider gate width of 200 μm with a unit finger width of 50 μm and four fingers, whose insertion loss is found to be 0.9 dB. Only M_5b_ for stage 5 is set to 100 μm with a unit finger width of 50 μm and two fingers, whose insertion loss is found to be 1.7 dB.

We also characterize the switch FET characteristics by finding the on-state resistance R_on_ and off-state capacitance C_off_. By simulations, R_on_ and C_off_ are found to be 21.3 Ω and 30.5 fF for 100 μm FET, 14.4 Ω and 45.6 fF for 150 μm FET, and 10.6 Ω and 55.9 fF for 200 μm FET. As a result, it is interesting to note that the figure-of-merits (FoMs) R_on_ × C_off_ fall in the range of 590–650 fsec for all switch FETs, which should guarantee the switching transient characteristics are almost the same for the phase shifter circuit.

**Figure 2:**
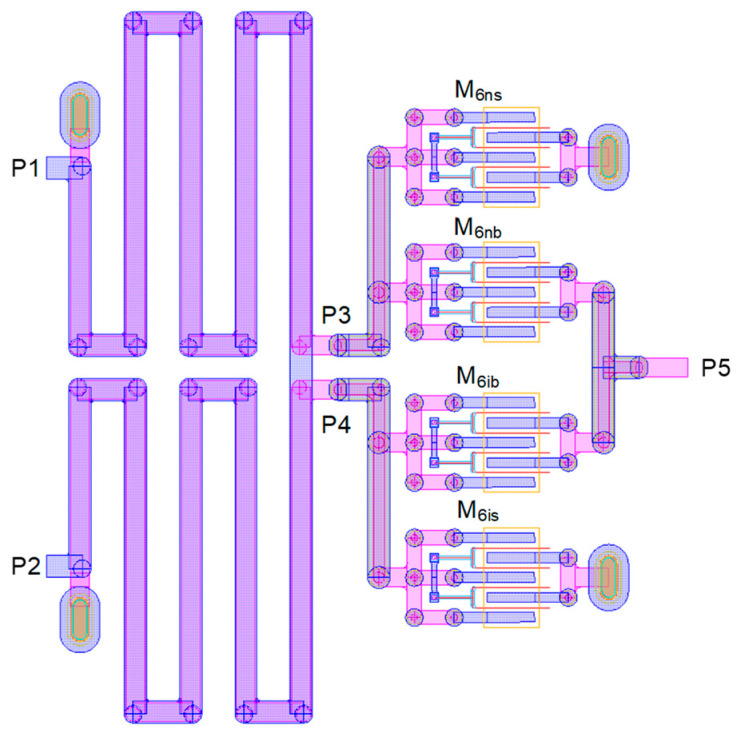
Layout of stage 6 comprising Marchand balun and SPDT switch.

Figure 2 exhibits the detailed layout design of stage 6. The Marchand balun is formed by stacking two metal layers given by the process technology. Its four ports are P_1_, P_2_, P_3_, and P_4_, in which P_1_ is the input port, P_2_ is open-ended, P_3_ is the non-inverting output port, and P_4_ is the inverting output port. The SPDT switch comprising M_6nb,6ns_ and M_6ib,6is_ turns on either the P_3_-to-P_5_ or P_4_-to-P_5_ path. The total routing length of the Marchand balun from P_1_ to P_2_ is 3.0 mm, which is much longer than the theoretical half wavelength because the many meandering effects are compensated. Full-wave electromagnetic (EM) field simulations are carried out for the layout design.

**Figure 3:**
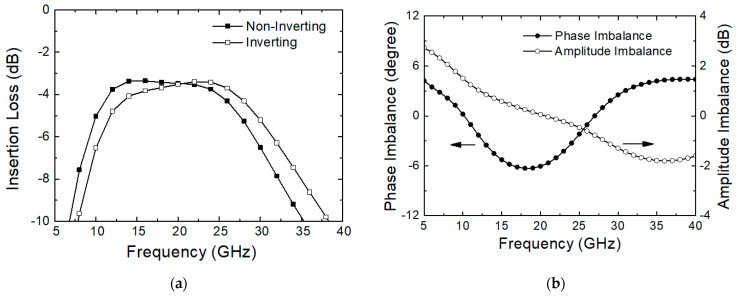
Simulated performance of stage 6. (**a**) Insertion loss; (**b**) phase and amplitude imbalance.

Figure 3a depicts the simulated insertion loss of stage 6. The simulations are carried out by including the EM effects of the Balun and routings as well as the FET models. The insertion loss at 28 GHz is 5.3 and 4.3 dB for the non-inverting (with M_6nb_ on, M_6ns_ off, M_6ib_ off, M_6is_ on) and inverting (with M_6nb_ off, M_6ns_ on, M_6ib_ on, M_6is_ off) state, respectively. It implies that the amplitude balance is well obtained for both states. Figure 3b illustrates the phase and amplitude imbalances between the inverting and non-inverting states. It shows that the imbalances at 28 GHz are lower than 1.5° for the phase and 1.1 dB for the amplitude. It also implies that the 180° phase shifting stage 6 provides well-balanced transfer characteristics of the amplitude and phase at both states.

## 3. Proposed Continuous Phase Calibration Technique

The proposed continuous phase-tuning calibration technique is implemented in stage 6. It is well known that the off-state capacitance of a switch FET is tunable by controlling the gate bias voltage. We can find that a similar approach has been adopted for X- and W-band GaN reflective-type phase shifter MMICs [17,18,19]. The reflective-type phase shifter simply adopts a tunable reactive component to tune the phase over a wide tuning range. In [17,18,19], we can find that they employ an off-state FET as a variable reactive load to tune the phase over 70° to 165°. However, they do not apply the technique for the fine phase calibration to reduce the phase error like this work.

In this work, we adopt the similar continuous tuning approach for the phase error calibration. Since any switch FETs can be adopted for this purpose, we need to decide which FETs are the best for the continuous phase-tuning calibration in this circuit. First, we find that the switch FETs in stages 1–5 cannot be utilized for the continuous tuning purpose. They will induce unacceptable variations at the overall phase shift performances. However, since stage 6 presents symmetric and balanced tuning characteristics regardless of its state, we can employ the stage-6 switch FET to tune the gate bias voltage for the continuous tuning purpose regardless of stage 6′s state.

**Figure 4:**
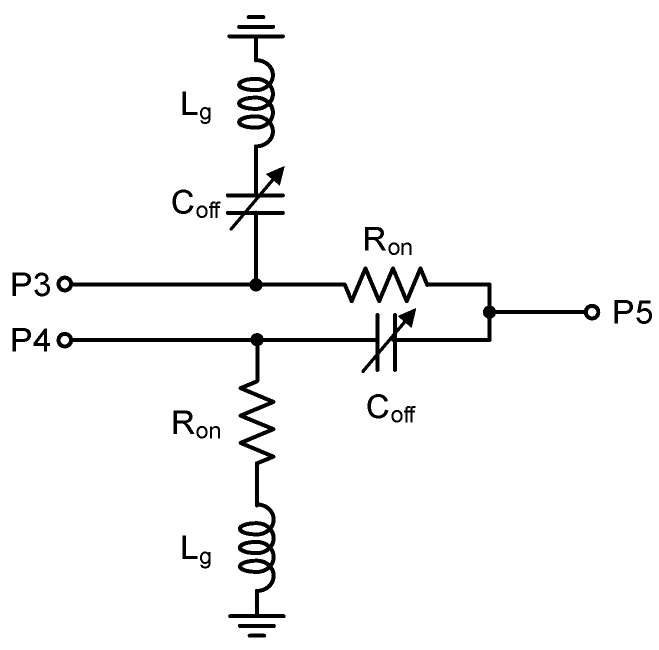
Equivalent circuit of the SPDT switch of stage 6.

Figure 4 is the equivalent circuit of the SPDT switch of stage 6, assuming the non-inverting path is turned on. The port numbers correspond to those shown in Figure 2. As can be seen, the SPDT imposes two shunt C_off_ values and two R_on_ values. The inductor L_g_ indicates a tiny parasitic inductance created by a short ground-access routing and back-side, which show that L_g_ is about 200 pH via EM simulations. For the sake of simplicity and clarity, we neglect R_on_ for the following theoretical analysis.

**Figure 5:**
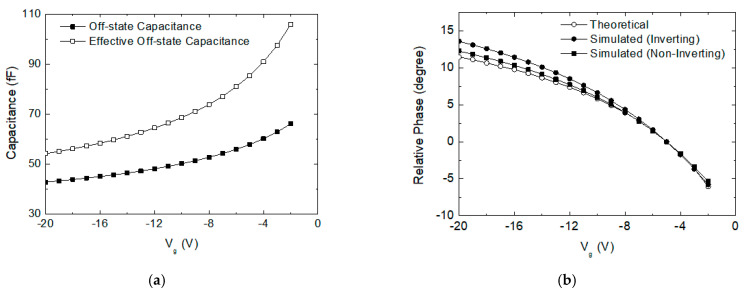
(**a**) Off-state capacitance with respect to the off-state FET’s gate voltage; (**b**) phase-tuning characteristics at the inverting and non-inverting states with respect to the off-state FET’s gate voltage.

First, by circuit simulations, we find the off-state capacitance C_off_ for the switch FET with respect to V_g_ between −2 and −20 V. Note that V_g_ must not be set higher than −2 V since it cannot be over the FET’s pinch-off voltage. Figure 5a shows that C_off_ with respect to V_g_ is tuned in 43–66 fF. Next, we compute an effective off-state capacitance $C_{off}^{\prime }$ given by the series connected C_off_ and L_g_. Note that $C_{off}^{\prime }$ can be written by

(1) $$C_{off}^{\prime }=\frac{C_{off}}{1-\omega^{2}L_{g}C_{off}}.$$

In Figure 5a, the computed $C_{off}^{\prime }$ by (1) is found to be tuned over 54–106 fF. Then, the theoretical insertion phase of Figure 4 can be written as

(2) $$\phi (S_{53})=-\tan^{-1}\left(\frac{\omega C_{off}^{\prime }R_{ο}}{2}\right),$$

where R_o_ is the source and load impedance of 50 Ω connected at P_3_ and P_5_ in Figure 4. The theoretical phase value computed by (2) is plotted in Figure 5b. Also, the simulated insertion phases for stage 6 at both phase-shifting states are also plotted in Figure 5b.

We can notice that the simulated phase characteristics agree very well with the theoretical computations. The results clearly show that the proposed tuning method can cover a wide phase-tuning range of 18° in a very fine step. We can also find that the continuous phase-tuning performances for both the inverting and non-inverting states are satisfactorily symmetric and balanced. It implies that the proposed continuous tuning calibration technique can effectively compensate for the phase error and improve the phase accuracy in the phase shifter.

**Figure 6:**
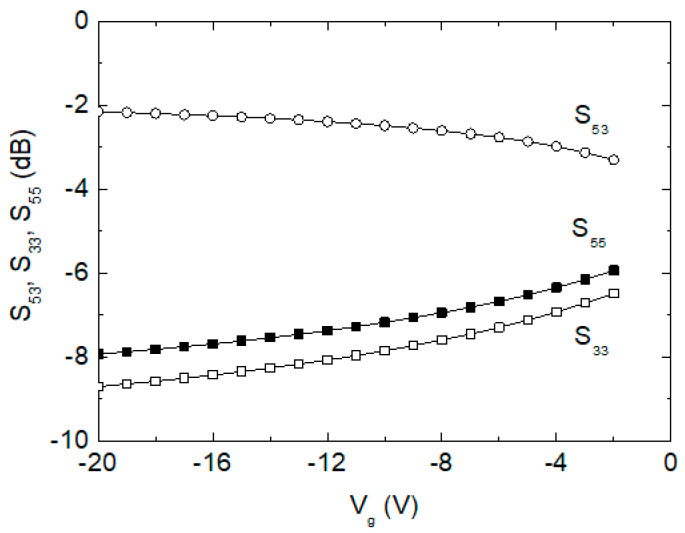
Simulated S_53_, S_33_, and S_55_ of the SPDT against V_g_.

We examine how the off-state gate voltage affects the insertion loss S_53_ and return losses S_33_ and S_55_ in the SPDT switch of Figure 2, and the results are shown in Figure 6. When V_g_ is tuned from −2 to −20 V, it is observed that S_53_, S_33_ and S_55_ change by 0.9 dB, 1.9 dB, and 2.2 dB, respectively. Thus, we can find that the V_g_ tuning imposes an insignificant impact on the phase shifter’s overall performances, which is also anticipated by the theoretical relations of S_53_ in (3) and S_33_ and S_55_ in (4). Note that (3) and (4) can be derived from the equivalent circuit of Figure 4.

(3) $$S_{53}=\frac{2}{2+R_{ο}\cdot j\cdot \omega \cdot (2{C^{\prime }}_{off})},$$

(4) $$S_{33}=S_{55}=\frac{-R_{ο}\cdot j\cdot \omega \cdot (2{{\cdot C}^{\prime }}_{off})}{2+R_{ο}\cdot j\cdot \omega \cdot (2\cdot {C^{\prime }}_{off})}.$$

## 4. Results and Discussion

**Figure 7:**
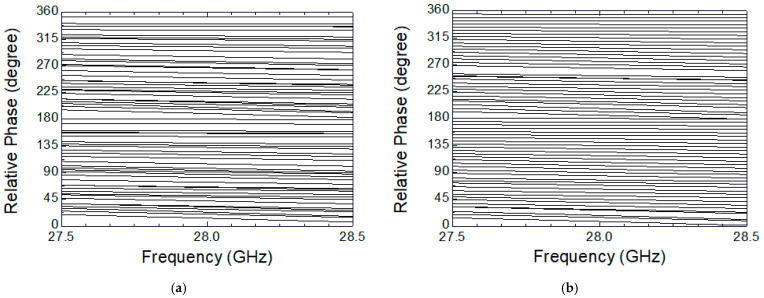
Simulated results of the phase shift characteristics. (**a**) Before calibration; (**b**) after calibration.

Full-3D EM simulations are carried out for the designed 6-bit phase shifter MMIC in order to examine overall phase shift characteristics, and these are shown in Figure 7. Figure 7a shows the 64-state phase shift characteristics without employing any calibration, and Figure 7b shows the same characteristics after applying the proposed calibration. Note that the 64 curves correspond to the 64 phase-shifting states that are set by the six-bit control word. The 64 curves comprise state 0 (0°) through state 63 (354.375°) with a step of 5.625°. As can be clearly seen, the irregular and uneven spacings between the 64 curves before calibration are dramatically improved to become evenly spaced and distributed after the calibration.

**Figure 8:**
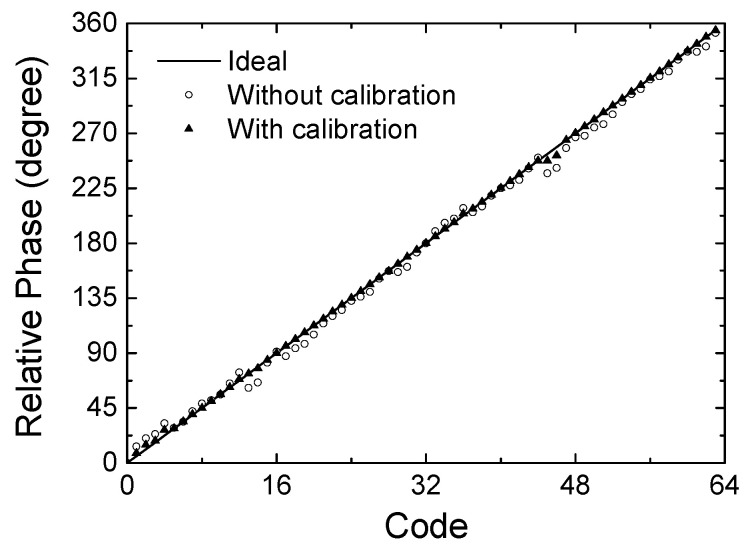
Simulated results of the phase shift characteristics with respect to 64 state codes.

Figure 8 redraws the relative phase with respect to the 64 phase state codes. The ideal curve is an ideal phase response with respect to the 64 digital phase state codes. The phase responses with and without the calibration are plotted together. The peak phase error is observed to be 17.1° at code 46 before calibration, and it is found to be reduced to 6.9° after calibration. These results clearly exhibit that the proposed calibration technique effectively improves the phase-tuning accuracy.

The designed circuit is fabricated in a commercially available 0.15 μm GaN HEMT process. The process offers a depletion-mode HEMT device with a pinch-off voltage of −2 V and a supply voltage of 28 V, two metallization layers with thicknesses of 3.1 and 3.3 μm, MIM (Metal–Insulator–Metal) capacitors with 0.18 fF/mm^2^, backside via with a 16 × 40 μm^2^ opening dimension, and a wafer thickness of 100 μm.

**Figure 9:**
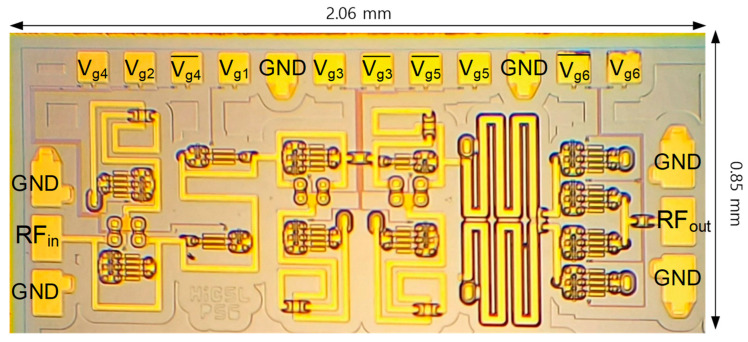
Chip micrograph.

Figure 9 shows a micrograph of the fabricated chip. The die area is 1.75 (=2.06 × 0.85) mm^2^. The chip is tested by using on-wafer G-S-G RF probes for the RF input and output signals and 12-pin dc probe for the dc supply and gate voltages. Note that the pads are denoted according to their circuit functions with regard to Figure 1.

**Figure 10:**
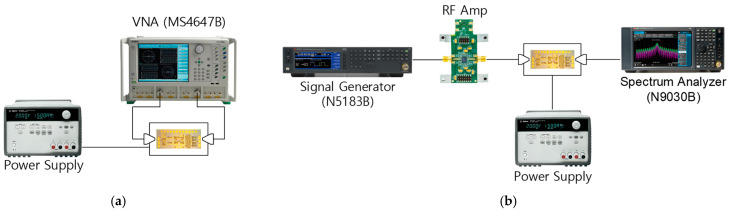
Measurement setup. (**a**) Small-signal characteristics; (**b**) large-signal characteristics.

The small- and large-signal measurement setups are illustrated in Figure 10. The small-signal performances of the phase shifter are measured by using an Anritsu MS4647B Vector Network Analyzer, and large-signal performances are measured by using a Keysight N9030B Signal Analyzer.

**Figure 11:**
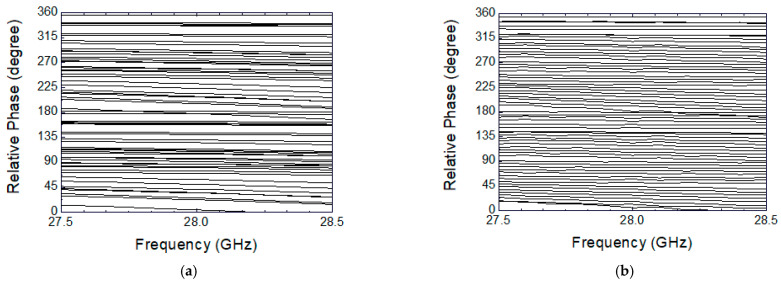
Measured phase shift characteristics. (**a**) Before calibration; (**b**) after calibration.

Figure 11 shows the measured phase response across 27.5–28.5 GHz. As also observed at the simulated characteristics in Figure 7a,b, the measured results also demonstrate that the phase shift characteristics before calibration are very irregular and uneven, and they dramatically improved after calibration.

**Figure 12:**
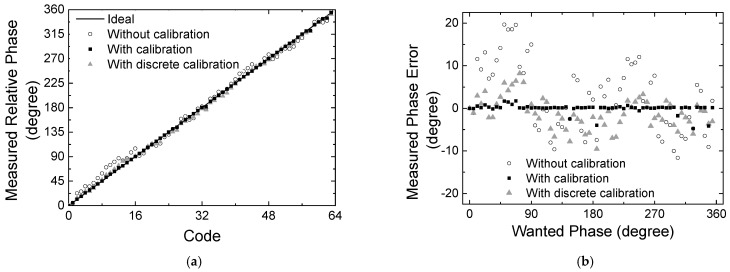
Measured phase shift characteristics with respect to the calibration methods. (**a**) Measured phase with respect to 6-bit 64 state codes; (**b**) spot phase errors.

Figure 12a,b redraw the measured phase transfer characteristics observed in Figure 11 at 28 GHz. Figure 12a shows the relative phase transfer characteristics across 64 codes, and Figure 12b shows the spot phase error at 64 individual states.

In addition to the proposed continuous calibration, we also employ a modified calibration technique as noted by a discrete calibration in Figure 12. The discrete calibration is to apply only 3-step V_g_ voltages of −3, −5, and −12 V instead of the continuous V_g_ voltage. It will make the calibration process easier at the cost of a slight degradation of the calibration accuracy. In Figure 12a,b, it is observed that the peak phase error of 19.6° before calibration is improved to 9.53° by the discrete calibration, and it further improved to 4.75° by the proposed continuous calibration.

**Figure 13:**
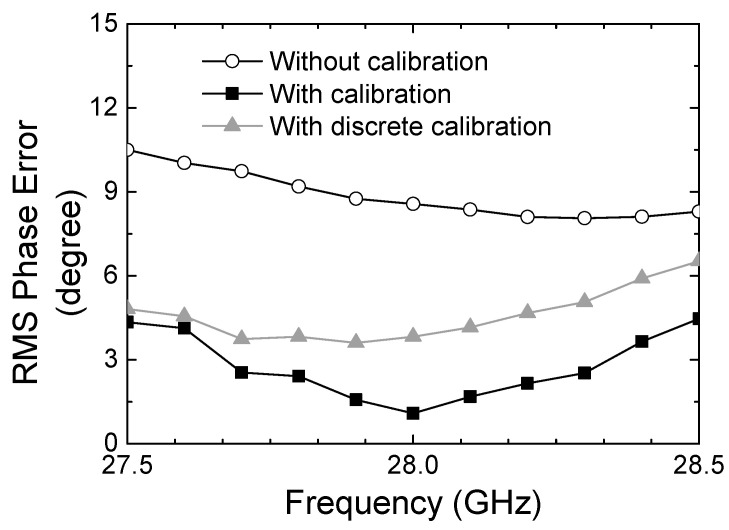
Measured RMS phase error with respect to the calibration methods.

Figure 13 illustrates the RMS phase error. At 28 GHz, it is 8.56° without calibration, while it improved to 3.82° by the discrete calibration, and it further improved to 1.08° by the continuous calibration. The measured results prove that the proposed calibration technique can significantly improve the phase-tuning accuracy.

**Figure 14:**
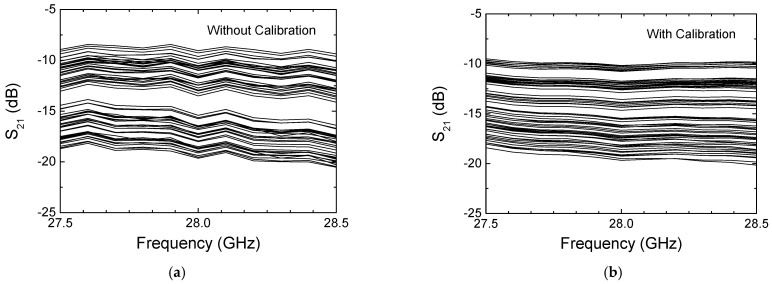
Measured insertion loss. (**a**) Without calibration; (**b**) with the proposed continuous calibration.

**Figure 15:**
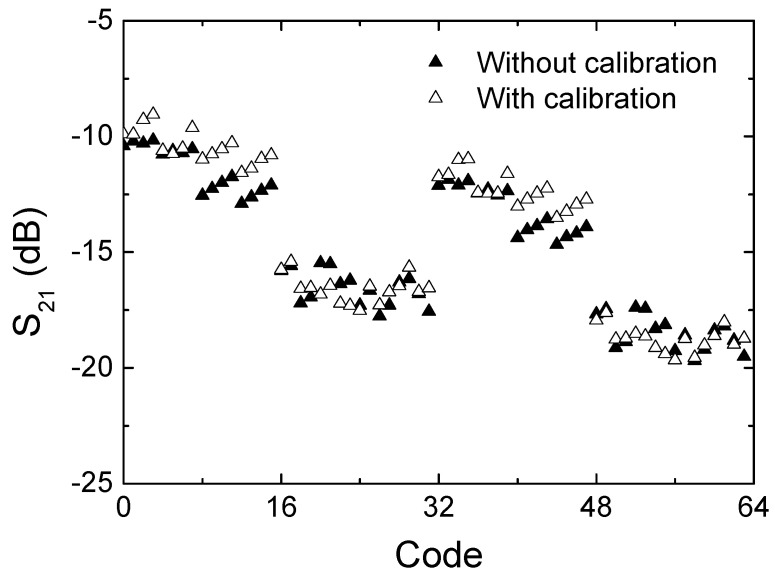
Measured insertion loss with respect to the 6-bit 64 states at 28 GHz.

Figure 14a,b show the measured insertion loss with and without calibration, respectively. It is observed that the insertion loss is within the range of 9.5 to 20 dB across the operating frequency band. In addition, Figure 15 more clearly compares the insertion loss at 28 GHz across the 64 codes. As can be seen, the insertion loss is 10.1–19.7 dB before the calibration and 9.1–19.7 dB after the calibration at 28 GHz. The results imply that the proposed calibration does not significantly change the insertion loss performances.

**Figure 16:**
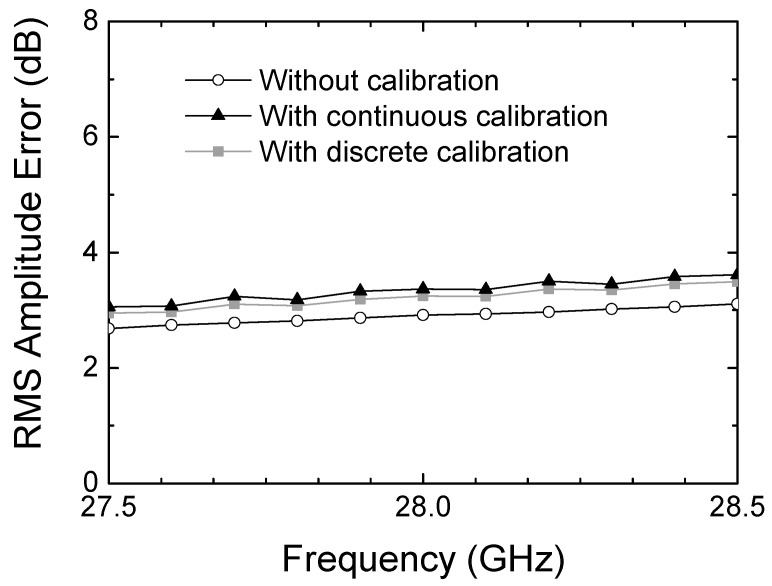
Measured RMS amplitude error.

The measured RMS amplitude error is illustrated in Figure 16. It is observed that the RMS amplitude error is 2.9 dB before calibration, 3.2 dB after the discrete calibration, and 3.3 dB after the continuous calibration. Although the slight degradation is observed, it is found to be not significant for the overall performances.

**Figure 17:**
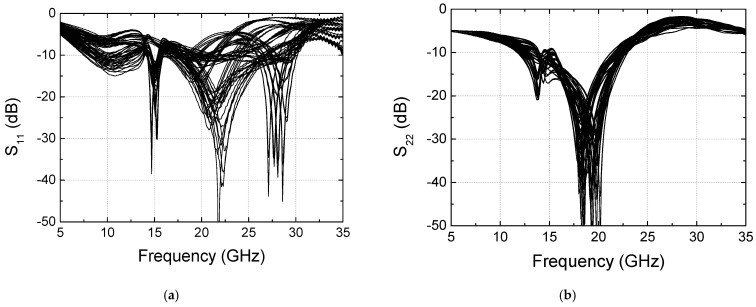
Measured return losses for 6-bit 64 states. (**a**) Input return loss S_11_; (**b**) output return loss S_22_.

We have measured the input and output return losses S_11_ and S_22_ for the 64 states, and the results are shown in Figure 17. Since we have observed that the calibration does not induce any significant changes in return losses, only the results after calibration are shown. At the operating frequency of 28 GHz, S_11_ is found to be −2 to −31 dB, and S_22_ is found to be −2 to −5 dB. Also, the best matched frequency band is found to be shifted down to around 20 GHz. Although the optimal return loss characteristics are not achieved, we have found that they can be further improved by carefully designing the input and output impedances while not significantly altering the phase-shift performances.

**Figure 18:**
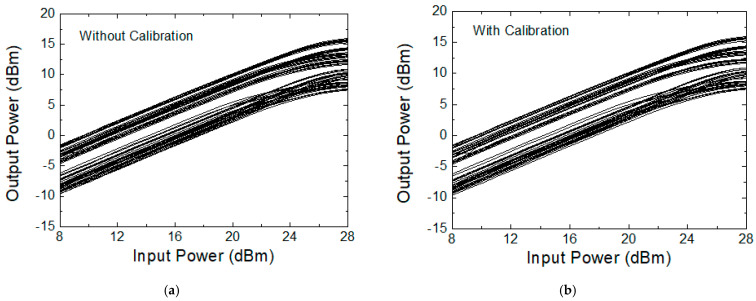
Measured input-to-output power transfer characteristics across the 64 states. (**a**) Without calibration; (**b**) with calibration.

**Figure 19:**
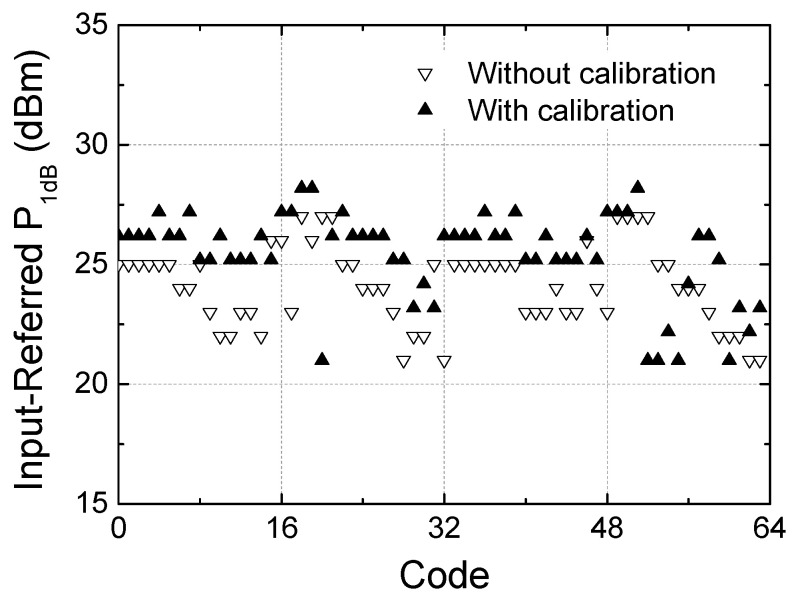
Measured input-referred P_1dB_ at 28 GHz against the 64 phase states.

Figure 18 illustrates the measured input-to-output power transfer characteristics across the 64 states. Figure 18a shows the characteristics before calibration, while Figure 18b shows those after calibration. Figure 19 illustrates the input-referred P_1dB_ at 28 GHz with and without calibration. The input-referred P_1dB_ is observed to be +21 to +27 dBm before the calibration and +21 to +28 dBm after the calibration, which also proves that the calibration does not induce any significant change in P_1dB_ performances. The input-referred P_1dB_ may indicate the maximum input power level that the phase shifter can handle without inducing significant signal distortions.

**Figure 20:**
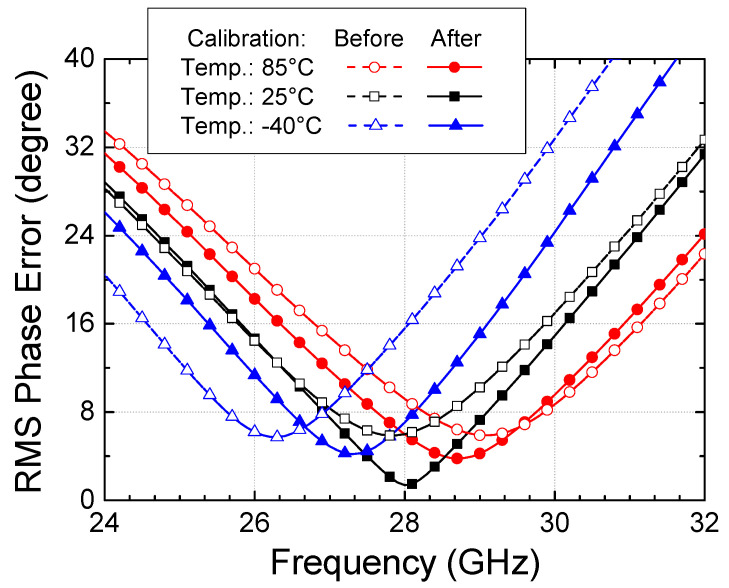
Temperature dependence of the phase shifter and calibration technique.

Finally, we have examined the temperature variation effects of the calibration technique. All the simulated and measured results are obtained at +25 °C; thus, we have carried out additional simulations to examine the phase shift responses at the low temperature of −40 °C and high temperature of +85 °C. As can be seen in Figure 20, the minimum RMS phase error at 25 °C is found to be 5.8° at 27.8 GHz before calibration and improved to 1.4° at 28 GHz after calibration. At −40 °C, the minimum RMS phase error is found to be 5.7° at 26.3 GHz before calibration and improved to 4.2° at 27.2 GHz after calibration. At +85 °C, the minimum RMS phase error is found to be 5.9° at 29 GHz before calibration and improved to 3.7° at 28.7 GHz after calibration. Thus, we can observe that the proposed calibration technique works well at the temperature corners, although the calibration becomes slightly less effective. Thus, we can conclude that the proposed calibration technique remains effective in a wide temperature range.

The performances of this work are summarized in Table 1. It comprises six stages producing a 6-bit 64-state phase shift with a minimum phase step size of 5.625°. The RMS phase error of this work is 8.6° before calibration, and it improved to 3.8° after the three-step discrete calibration and further improved to 1.08° after the proposed continuous calibration. Also, note that the improvement of the phase error is achieved without any critical degradation in other circuits performances such as insertion loss and input-referred P_1dB_, which are 10.1–19.7 dB and 21–28 dBm, respectively. The RMS amplitude error is 2.9 dB. The die area is 1.75 mm^2^ and the process uses 0.15 μm GaN HEMT technology.

This work is compared to other recent works in Table 1. Although we cannot find the same frequency band and same resolution digital GaN phase shifter MMICs, we have chosen the most recent state-of-the-art digitally controlled phase shifter MMICs with a rather wide variation in the process technology and frequency band. In terms of process technology, Refs. [6,7,8,9,10] used GaN, Ref. [14] used GaAs, and [13,15] used CMOS. And in terms of frequency band, Ref. [15] operates at the same frequency band with this work at 28 GHz, whereas [6,7,8,13] operate in the X band, Ref. [14] operate at 2–4 GHz, and [9,10] operate in the Ka band. It should be pointed out that none of the previous GaN digital phase shifters [6,7,8,9,10] had employed a calibration technique, thus inevitably resulting in a relatively higher phase error. Even the most recent 6-bit GaN phase shifter MMICs [9,10] did not employ any calibration technique, and thus the RMS phase error was a bit mediocre 4–6°.

The CMOS and GaAs digital phase shifters of [13,14,15] employ the conventional LSB code calibration to obtain a better accuracy. Refs. [13,14] utilized one LSB for their LSB code calibration, and [15] utilized three LSBs for the LSB code calibration. However, it is found that their phase error improvement was quite limited. For example, Ref. [13] showed that the initial phase error was 8° before calibration and improved only to 6° after the LSB calibration.

As can be noted, this work produces the best RMS phase error of 1.08° by employing the proposed calibration technique. It implies that the proposed technique is instrumental for millimeter-wave digital phase shifter MMICs.

## 5. Conclusions

In this work, we have presented a 6-bit digital phase shifter MMIC in a 0.15 μm GaN HEMT process for Ka-band phased-array transceiver systems for millimeter-wave wireless communication and sensing applications. The circuit comprises the switched-line and switched-filter circuits for the first through fifth stages as well as a Marchand balun combined with an SPDT switch for the final sixth stage. The off-state capacitance of the SPDT switch at stage 6 is either continuously or three-step discretely tuned for fine phase-tuning calibration. We have achieved the RMS phase error of 1.08° at 28 GHz by applying the proposed continuous phase calibration technique. Compared to the conventional LSB code calibration, this technique more effectively improves the phase error while not consuming additional circuitry. The simulated and measured results confirm the effectiveness of the continuous phase calibration technique, showing not only improved phase accuracy but also maintaining desirable characteristics such as the insertion loss and input-referred P_1dB_. The proposed continuous technique can be instrumental for a millimeter-wave multi-bit digital phase shifter MMIC design, offering superior performance in terms of phase error, phase shift resolution, and chip area.

## Figures and Tables

**Table 1:** Performance summary and comparison.

	This Work			[10]	[9]	[8]	[7]	[6]	[14]	[13]	[12]
Frequency (GHz)	28			37–40	36–39	8–10	8–12	8–12	27–29.5	2.4–4	8–12
Phase-Tuning Calibration Method	None	Proposed discrete calibration	Proposed continuous calibration	None	None	None	None	None	3-LSB	1-LSB	1-LSB
Number of Stages	6			6	6	3	5	5	9	8	7
Phase Shift Resolution (bit)	6			6	6	3	5	5	6	7	6
Phase Step Size (°)	5.625			5.625	5.625	45	11.25	11.25	5.625	2.8	5.625
RMS Phase Error (°)	8.6	3.8	1.08	5.36	4.6	3 ^†^	2.5–6.2	6.4	3.5	1.5	6
RMS Amplitude Error (dB)	2.9	3.2	3.4	3.21	0.62	1.1	1.2	0.8	0.4	0.34	0.45
Insertion Loss (dB)	10.1–19.7	9.2–20	9.5–19.7	10.5	8.7	12–15	8.2–15.1	14	-	4.9	15
Input-Referred P_1dB_ (dBm)	21–28			10–16 *	27.7	32	29	34.8	-	29.6	11
Chip Size (mm^2^)	1.75			3.36	3.67	10.25	6	23.5	1.31 ^#^	1.83	1.2
Process	0.15 µm GaN			0.15 µm GaN	0.15 µm GaN	0.25 µm GaN	0.25 µm GaN	0.25 µm GaN	65 nm CMOS	0.5 µm GaAs	0.13 µm CMOS

^†^ Peak error, ^#^ Estimated, * Simulated result.

## Data Availability

Data are contained within the article.
